# Seroprevalence of exposure to SARS-CoV-2 in domestic dogs and cats and its relationship with COVID-19 cases in the city of Villavicencio, Colombia

**DOI:** 10.12688/f1000research.125780.1

**Published:** 2022-10-17

**Authors:** Dumar Alexander Jaramillo Hernández, María Clara Chacón, María Alejandra Velásquez, Adolfo Vásquez-Trujillo, Ana Patricia Sánchez, Luis Fabian Salazar Garces, Gina Lorena García, Yohana María Velasco-Santamaría, Luz Natalia Pedraza, Lida Carolina Lesmes-Rodríguez

**Affiliations:** 1 Escuela de Ciencias Animales, Universidad de los Llanos, Villavicencio, Meta, 1745, Colombia; 2 Programa de Medicina Veterinaria y Zootecnia, Escuela de Ciencias Animales, Facultad de Ciencias Agropecuarias y Recursos Naturales, Universidad de los Llanos, Villavicencio, Meta, 1745, Colombia; 3 Secretaria de Salud Municipal, Alcaldía de Villavicencio, Villavicencio, Meta, 110221, Colombia; 4 Research and Development Department (DIDE), Faculty of Health Sciences, Technical University of Ambato, Ambato, Ambato, Av. Colombia and Chile s/n, Ecuador; 5 Departamento de Biología & Química, Facultad de Ciencias Básicas e Ingeniería, Universidad de los Llanos, Villavicencio, Meta, 1745, Colombia

**Keywords:** anthropozoonosis, coronavirus, immunoassay, public health

## Abstract

**Background**: Since the beginning of the severe acute respiratory syndrome coronavirus 2 (SARS-CoV-2) outbreak, different animal species have been implicated as possible intermediate hosts that could facilitate the transmission of the virus between species. The detection of these hosts has intensified, reporting wild, zoo, farm, and pet animals. The goal of this study was to determine the seroprevalence of anti-SARS-CoV-2 immunoglobulins (IgG) in domestic dogs and cats and its epidemiological association with the frequency of coronavirus disease 2019 (COVID-19) patients in Villavicencio, Colombia.

**Methods:** 300 dogs and 135 cats were randomly selected in a two-stage distribution by clusters according to COVID-19 cases (positive RT-qPCR for SARS-CoV-2) within the human population distributed within the eight communes of Villavicencio. Indirect enzyme-linked immunosorbent assay (ELISA) technique was applied in order to determine anti-SARS-CoV-2 IgG in sera samples. Kernel density estimation was used to compare the prevalence of COVID-19 cases with the seropositivity of dogs and cats.

**Results:** The overall seroprevalence of anti-SARS-CoV-2 IgG was 4.6% (95% CI=3.2-7.4). In canines, 3.67% (95% CI=2.1-6.4) and felines 6.67% (95% CI=3.6-12.18). Kernel density estimation indicated that seropositive cases were concentrated in the southwest region of the city. There was a positive association between SARS-CoV-2 seropositivity in pet animals and their habitat in Commune 2 (adjusted OR=5.84; 95% CI=1.1-30.88). Spearman's correlation coefficients were weakly positive ( *p*=0.32) between the ratio of COVID-19 cases in November 2020 and the results for domestic dogs and cats from the eight communes of Villavicencio.

**Conclusions:** In the present research cats were more susceptible to SARS-CoV-2 infection than dogs. This study provides the first positive results of anti-SARS-CoV-2 ELISA serological tests in domestic dogs and cats in Colombia with information about the virus transmission dynamics in Latin America during the COVID-19 pandemic.

## Introduction

Coronavirus disease 2019 (COVID-19) emerged in the Huanan Seafood Wholesale Market in Wuhan, China, in December 2019. ^1^ Severe acute respiratory syndrome coronavirus 2 (SARS-CoV-2) was the causal agent of this disease, which was declared as pandemic by the World Health Organisation (WHO) on 11 ^th^ March 2020. ^2^ The zoonotic origin of COVID-19 has been evidenced thanks to the high genomic similarity of SARS-CoV-2 with coronaviruses (CoV) recovered from bats ^3^ and pangolins, the latter considered potential intermediate hosts of the virus. ^4^ Betacoronaviruses such as SARS-CoV-2 belong to the family Coronaviridae. They exhibit linear single-stranded RNA of positive polarity ^5^ and cause respiratory and gastrointestinal diseases in mammals. ^6^ Even though humans are the most frequent route of transmission, it has been reported that cats ( *Felis catus*) and dogs ( *Canis lupus familiaris*) are susceptible to SARS-CoV-2 infection. ^7^ This way, reverse zoonosis (anthropozoonosis) is viable through close contact with owners during acute infections. ^8^

Since the beginning of the SARS-CoV-2 outbreak, different animal species have been implicated as possible intermediate hosts that could facilitate the virus transmission between species. ^9^ This is the reason why the determination of these hosts has intensified, evidencing a number of reports involving wild, zoo, farm and pet animals. ^10^ The zoonotic nature from which the transmission hypothesis has started, determines the importance of investigating animal species considered natural reservoirs of SARS-CoV-2. ^11^ However, concern for the control and reduction of the spread of the virus has led to more vigorous investigation of the role that pet animals, such as dogs and cats, play in the spread of the disease. Although a cat-to-human transmission case was reported, ^12^ it has been clearly defined that domestic canines and felines do not play a relevant role in the virus transmission to humans. ^8^^,^^13^

On the other hand, human-animal transmission has been widely reported. ^14^^,^^15^ This fact generates the need to investigate the implications for public and animal health, taking into account that animals are an epidemiological part of this pandemic. ^16^ Various epidemiological and experimental studies, through serological detection of antibodies against SARS-CoV-2, neutralising antibodies, and detection of viral genome by reverse transcriptase polymerase chain reaction (RT-qPCR), have confirmed SARS-CoV-2 in pet animals around the world. ^17^ Likewise, the occurrence of emerging variants has been described, as well as their influence on animals, ^18^ for example, the Alpha variant (B.1.1.7) in dogs and cats with clinical signs of myocarditis, ^19^ and the Delta variant (B.1.617.2) in dogs with clinical digestive and respiratory symptoms. ^20^ Regarding the Omicron variant (B.1.1.529), concluded that the SARS-CoV-2 virus accumulated mutations within host cells in mice, giving rise to the Omicron variant that was transmitted to humans, indicating a ‘ *ping-pong’* (spillover and spillback) evolutionary trajectory between species. ^21^ Transmission of SARS-CoV-2 Delta variant (AY.127) from hamsters to humans ^22^ and animal-to-human transmission of SARS-CoV-2 within mink farms ^23^ have also been reported.

Susceptibility to SARS-CoV-2 is determined by the affinity between the receptor-binding domain (RBD) of the viral spike (S) glycoprotein and the angiotensin-converting enzyme 2 (ACE2) of the host cell. Therefore, since vertebrates have conserved domains of ACE2, transmission of the virus between species becomes possible. ^24^ Canines have lower susceptibility to SARS-CoV-2 infection in contrast to felines ^8^^,^^25^ that exhibit greater respiratory pathology and efficient transmission of the virus to other felines through aerosols. ^26^

In the context of the rapid evolutionary trajectory between species that SARS-CoV-2 has been developing, and taking into account the Report No. 13 of the World Organisation for Animal Health (OIE) of 31 ^st^ May 2022, which reported 676 outbreaks in animals affecting 23 different species in 35 countries, ^27^ the need for seroepidemiological monitoring in pet, wild and synanthropic animals becomes essential in order to broadly understand the adaptation, evolution and transmission of SARS-CoV-2. ^7^

In Colombia, in December 2021, a lion exhibited symptoms of infection days after being in contact with a COVID-19 positive keeper. ^28^ However, seroepidemiological studies of exposure to SARS-CoV-2 by pet animals have not been reported to date in the country. The goal of the present study was to determine the seroprevalence of anti-SARS-CoV-2 immunoglobulins (Ig) class G (IgG) in domestic dogs and cats and its epidemiological association with the frequency of COVID-19 patients in Villavicencio city, Colombia.

## Methods

### Ethical considerations

This research was endorsed by the Bioethics Committee of the Universidad de los Llanos, according to Minute 02 by consensus of April 6, 2021. In addition, all the owners of the dogs and cats involved in this study signed the respective informed consent.

### Type of study and sample size

This is a cross-sectional epidemiological study conducted in Villavicencio, Colombia. It consisted of applying a characterisation survey and taking blood samples from domestic canines and felines. The sample was estimated using the formula for size by proportions in finite populations, using the results obtained by Patterson *et al*. ^29^ as a reference of *p*, with SARS-CoV-2 seroprevalence of 9.1% (3.3% in canines and 5.8% in felines) in pet animals in Italy. The population assessed in the present study corresponded to 68,651 domestic canines and felines in Villavicencio (47,573 canines and 21,078 felines), according to estimates from the report on anti-rabies vaccination of dogs and cats in Colombia. ^30^ The confidence interval (CI) considered was 95% and the ‘ *Z*’ value was 1.96 (1-α). The absolute precision considered was 0.15% (d = 0.0051).

### Sampling and inclusion criteria

**Figure 1.**
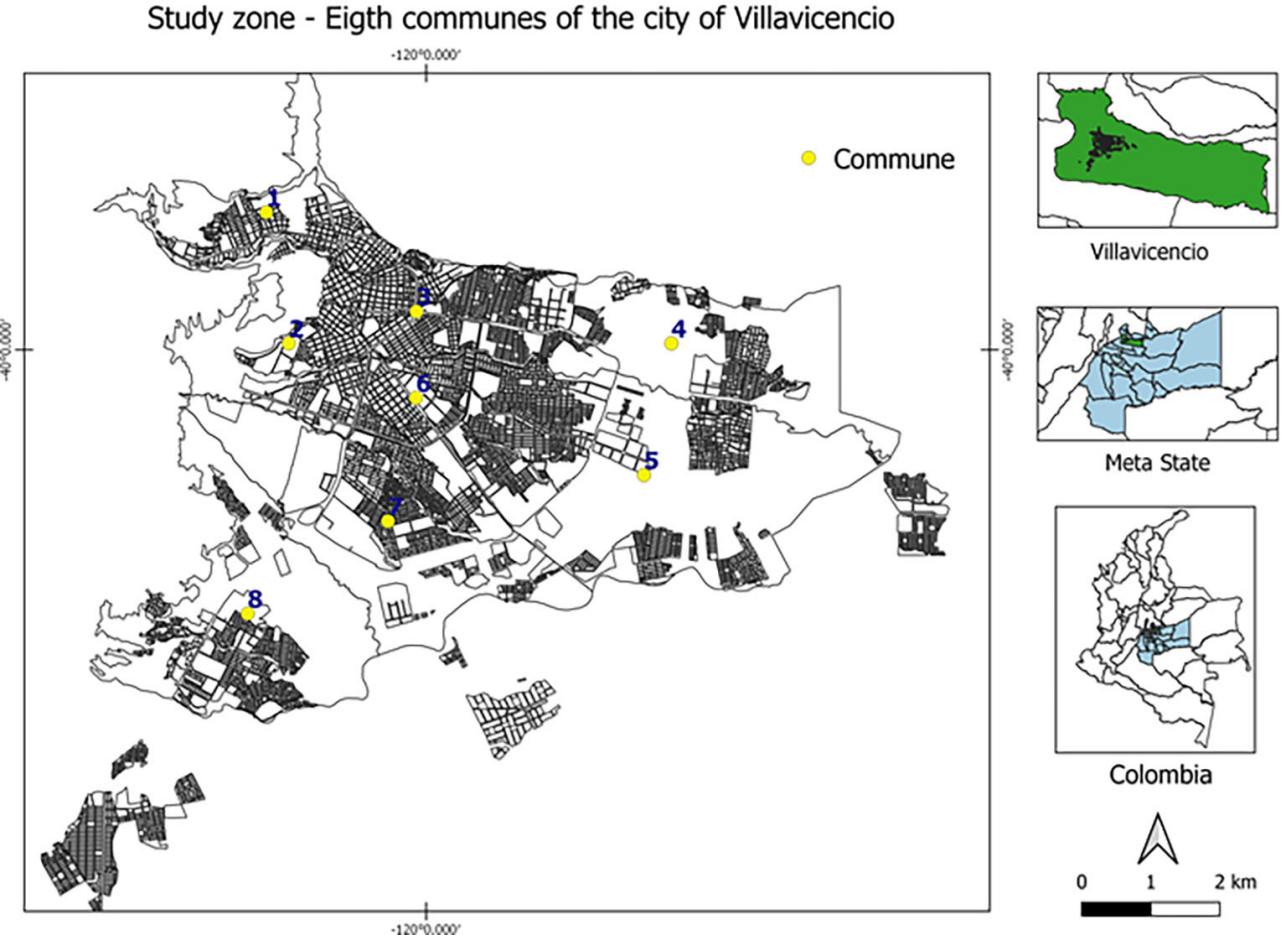
Study zone – eight communes of Villavicencio city, Meta state, Colombia. This figure is an original figure produced by the authors for this article.

The participants were selected based on their mandatory participation in the 2021 rabies vaccination campaign, carried out in Villavicencio (Meta State) by the health secretary. A probabilistic sampling was conducted by randomly selected two-stage clusters of domestic dogs and cats from the eight communes that compose the urban area of Villavicencio ( Figure 1), which consisted of the random and proportional selection of individual dogs and cats, the sampling proportion of each cluster was determined according to the frequency of COVID-19 cases (RT-qPCR testing) in each commune ^31^ (according to Table 1); for this, the EpiInfo v. 3.0 software, from the US Centers for Disease Control and Prevention (CDC) was used ( https://www.cdc.gov/epiinfo/esp/es_index.html). The inclusion criteria considered domestic dogs and cats that had lived constantly in their homes for a minimum of two months before starting the present study. The animals that had consumed immunomodulatory medication (e.g., corticosteroid-type immunosuppressants) one week before the sampling were not included in the study.

**Table 1.** Pet animal distribution among the eight communes of Villavicencio city according to coronavirus disease 2019 (COVID-19) active cases. Active human cases (RT-qPCR) in November 2020; data from the Villavicencio Municipal Health Secretary (2021).

Commune	COVID-19* active cases (%)	Dogs	Cats	Total pet animals
1	7.4	22	10	32
2	10	30	14	44
3	3.5	10	5	15
4	14.3	43	19	62
5	22.4	68	30	98
6	4.8	14	7	21
7	18.9	58	25	83
8	18.7	55	25	80
**Total**	100	300	135	435

A total of 435 blood samples were taken (300 domestic canines and 135 domestic felines). For this purpose, the authors of this study collected the blood from the jugular or cephalic vein, previous disinfection of the area with alcohol using a 21-gauge needle or vacutainer. Haemostasis was facilitated by applying pressure with sterile gauze in the sampling site for approximately 30 sec. The samples were centrifuged at 2000 g (Centrifuga Eppendorf 5424R) within three hours after being taken, and the sera were stored at -20 °C until analysis in a freezer (ABBA CVANF502B1). Table 1 shows the representative distribution of ‘n’ by commune (235 neighbourhoods) in Villavicencio.

Pet animals characterization was performed through a survey applied to the owners, following the model of a SARS-CoV-2 study that involved dogs and cats with COVID-19 patients in a metropolitan area. ^32^ The characteristics of each pet recorded were: name; sex; age; species; breed; and owners’ names. This survey also inquired whether the individuals that cohabited with the pet animals (spontaneous communication) had histories of positive or negative RT-qPCR testing for COVID-19, and whether there were histories of clinical signs of the animals, such as signs in the upper or lower respiratory tract, or non-specific digestive signs (e.g., vomiting, diarrhoea, among others). Coordinates of the houses where the pets lived, were also recorded. The survey can be found as *Extended data*. ^49^

### Immunoassay

IgG antibodies against the nucleocapsid protein (N) of SARS-CoV-2 in the sera of domestic dogs and cats were qualitatively determined using the indirect enzyme-linked immunosorbent assay (ELISA) (ID Screen ^®^ SARS-CoV-2, double antigen multi-species [IDvet, Grabels, France]) according to the manufacturer's instructions. Each plate contained 96 microwells sensitised with recombinant antigen of purified N protein of SARS-CoV-2, to which the following items were added: two negative controls (NC); two positive controls (PC); and 92 problem sera previously homogenised by vortexing. The optical density (OD) reading was performed using the Cytation 3 multimodal microplate reader (BioTek Instruments, Inc. Winooski, VT, USA) with a wavelength of 450 nm. In total, 435 problem sera samples and 25 pre-pandemic canine sera previously stored at -20 °C were analysed. Using the OD data of each well, the sample/positive control (S/P) ratio was calculated, which was expressed as a percentage using the following formula:

$$S/P\%=\frac{\mathrm{OD}\;\text{sample}-\mathrm{DO}\;\mathrm{NC}}{\mathrm{OD}\;\mathrm{PC}-\mathrm{OD}\;\mathrm{NC}}\times 100$$

The test was validated when the mean OD value of the PC was greater than 0.350, and the ratio of the mean OD values of the PC and NC was greater than three. The samples were considered positive if the S/P ratio was greater than or equal to 60%, doubtful samples or samples in the gray zone had S/P ratios between 50% and 60%, and samples with S/P ratio less than or equal to 50% were considered negative.

The step-by-step protocol of this trial has been deposited under the title: Immunoassay of SARS-CoV-2 in dogs and cats V.1, DOI: dx.doi.org/10.17504/protocols.io.5qpvorn29v4o/v1 in protocolos.io ( https://www.protocols.io/view/immunoassay-of-sars-cov-2-in-dogs-and-cats-v-1-5qpvorn29v4o/v1).

### Statistical analysis

The frequencies of the data obtained in the survey and transformation of quantitative variables into categories for their subsequent analysis were estimated. The punctual seroprevalence (P) of SARS-CoV-2 in pet animals in Villavicencio was expressed as a proportion using the following formula, considering 95% CI:

$$P\%=\frac{\#\text{of SARS}\;\mathrm{CoV}\;2\;\text{seropositive cases}}{\mathrm{sample}\;\mathrm{size}}\times 100$$

The risk association measure odds ratio (OR), calculated by the binomial logistic regression model with 95% CI; was used in order to determine whether the frequency of active COVID-19 cases in humans by commune was related to SARS-CoV-2 seropositivity (exposure) of pet animals. Likewise, Spearman correlation was used to establish a possible relationship in the increase of cases in domestic animals at homes with COVID-19. Finally, using Kernel density analysis, the prevalence of COVID-19 in humans by commune was compared to anti-SARS-CoV-2 IgG seropositivity in domestic dogs and cats. A confidence level of 95% was used for all statistical calculations. Statistical estimates were made using the R 4.2 software, and the maps using the QGIS 3.10 software.

## Results

**Figure 2.**
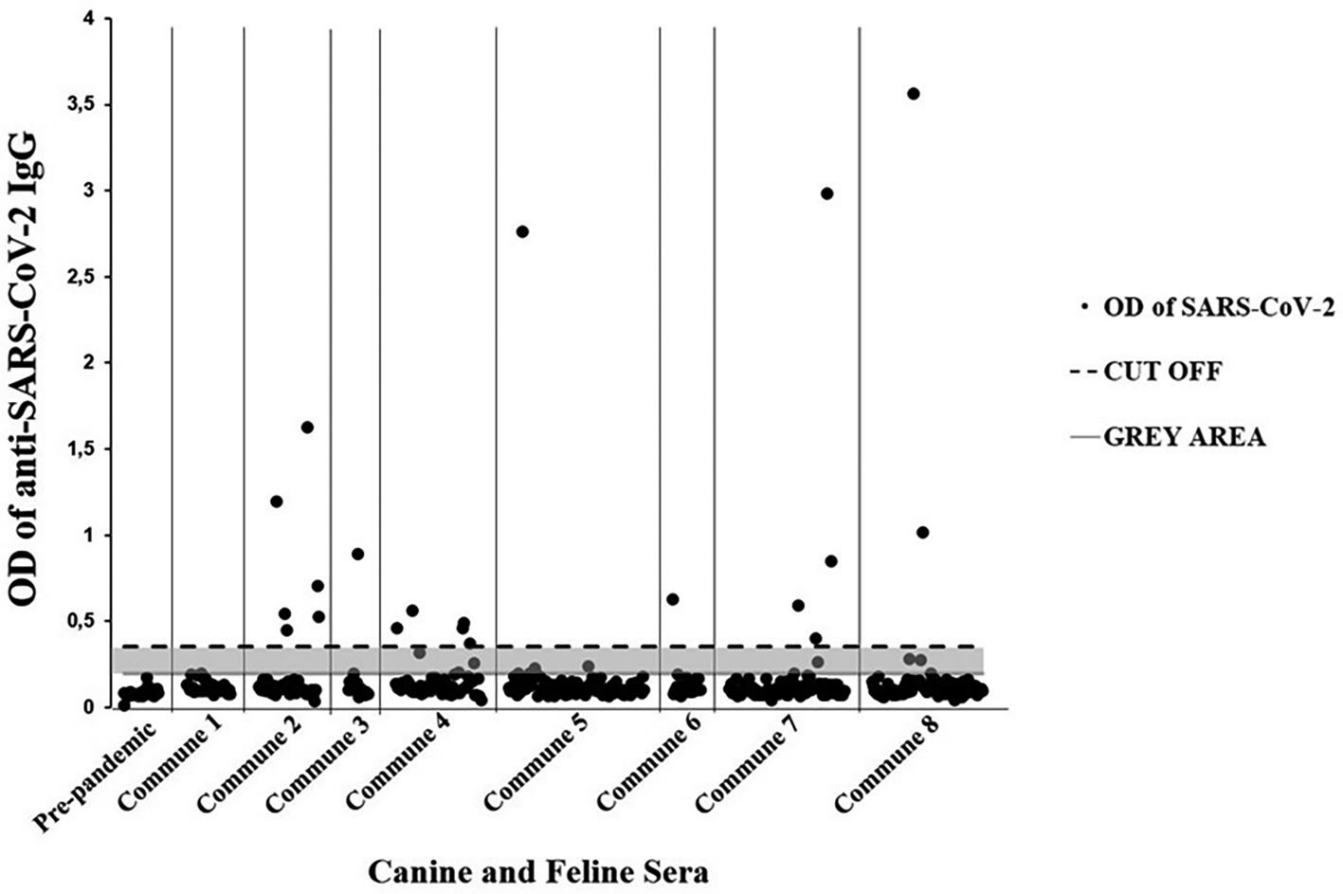
Domestic dogs and cats with SARS-CoV-2 seropositivity from the eight communes of Villavicencio. Ig = immunoglobulin class G; OD = optical density; SARS-CoV-2 = severe acute respiratory syndrome coronavirus 2.

The overall seroprevalence of anti-SARS-CoV-2 IgG was 4.60% (95% CI = 3.2-7.4). ^49^ Specifically, in canines the results indicated 3.67% (95% CI = 2.1-6.4), and in felines 6.67% (95% CI = 3.6-12.18) ( Table 2). Twenty seropositive individuals (11 canines and 9 domestic felines) were detected through the enzyme immunoassay. In general, 22 animals with a history of respiratory signs (e.g., cough, runny nose, among others) were detected, of which 9.10% (95% CI = 2.53-27.81) were seropositive for SARS-CoV-2 ( Table 3). Additionally, seven immunoassay results were classified as doubtful (gray area). Likewise, all 25 canine pre-pandemic sera were negative ( Figure 2).

**Table 2.** Characterisation of domestic canines and felines according to their SARS-CoV-2 seropositivity. The following variables were taken into account: species, age, city communes and owners with positive or negative RT-qPCR testing.

		n	%	SARS-CoV-2 seropositivity	Crude OR	95% CI	Adjusted OR	95% CI
**Species**	Canines	300	69	3.67% (11/300)	1	1	1	1
	Felines	135	31	6.67% (9/135)	1.87	0.76-4.64	2.07	0.78-5.46
**Age (Years)**	0-5	344	79	4.66% (16/344)	1	1	1	1
	6-10	78	18	3.85% (3/78)	0.85	0.24-2.98	0.92	0.25-3.46
	11-15	13	3	7.69% (1/13)	1.85	0.22-15.2	1.61	0.16-15.93
	1	32	7	0% (0/32)	1	1	1	1
	2	44	10	13.63% (6/44)	4.17	1.52-11.49	5.84 *	1.1-30.88
	3	15	3	6.67% (1/15)	1.48	0.18-11.86	1.97	0.15-25.69
	4	62	14	8.06% (5/62)	2.13	0.75-6.12	3.13	0.57-17.36
	5	98	23	1.02% (1/98)	0.17	0.02-1.29	0.37	0.03-4.2
	6	21	5	4.76% (1/21)	1.02	0.13-8.01	1.96	0.17-23.06
	7	83	19	4.82% (4/83)	1.07	0.35-3.3	2.02	0.36-11.45
	8	80	18	2.5% (2/80)	0.85	0.18-4.67	1.04	0.21-12.21
**Owners COVID 19**	COVID Test +	25	6	4% (1/25)	0.13	0.01-2.56	0.09	0-2.49
	COVID Test -	410	94	4.63% (19/410)	0.14	0.01-1.43	0.12	0.01-1.56

*Note.* 95% CI = confidence interval; COVID-19 = coronavirus disease 2019; OR = odds ratio; RT-qPCR = reverse transcriptase polymerase chain reaction; SARS-CoV-2 = severe acute respiratory syndrome coronavirus 2.

* *p* <0.05.

**Table 3.** Animals with a history of respiratory and digestive signs related to SARS-CoV-2 seropositivity.

		No.	%	SARS-CoV-2 seropositivity	95% CI	*X* ^2^	*p*-value
**Animals with respiratory signs history**	Exhibited signs	22	5.1	9.10% (2/22)	2.53-27.81	0.8206	0.365
	Dis not exhibit signs	413	94.9	4.56% (18/395)	2.90-7.09		
**Animals with digestive signs history**	Exhibited signs	9	2.1	0% (0/9)	0-29.91	NA	NA
	Dis not exhibit signs	426	97.9	4.93% (20/406)	3.21-7.49		

*Note.* 95% CI = confidence interval; NA = no results are presented because there were no pets detected with digestive signs; SARS-CoV-2 = severe acute respiratory syndrome coronavirus 2.

**Figure 3.**
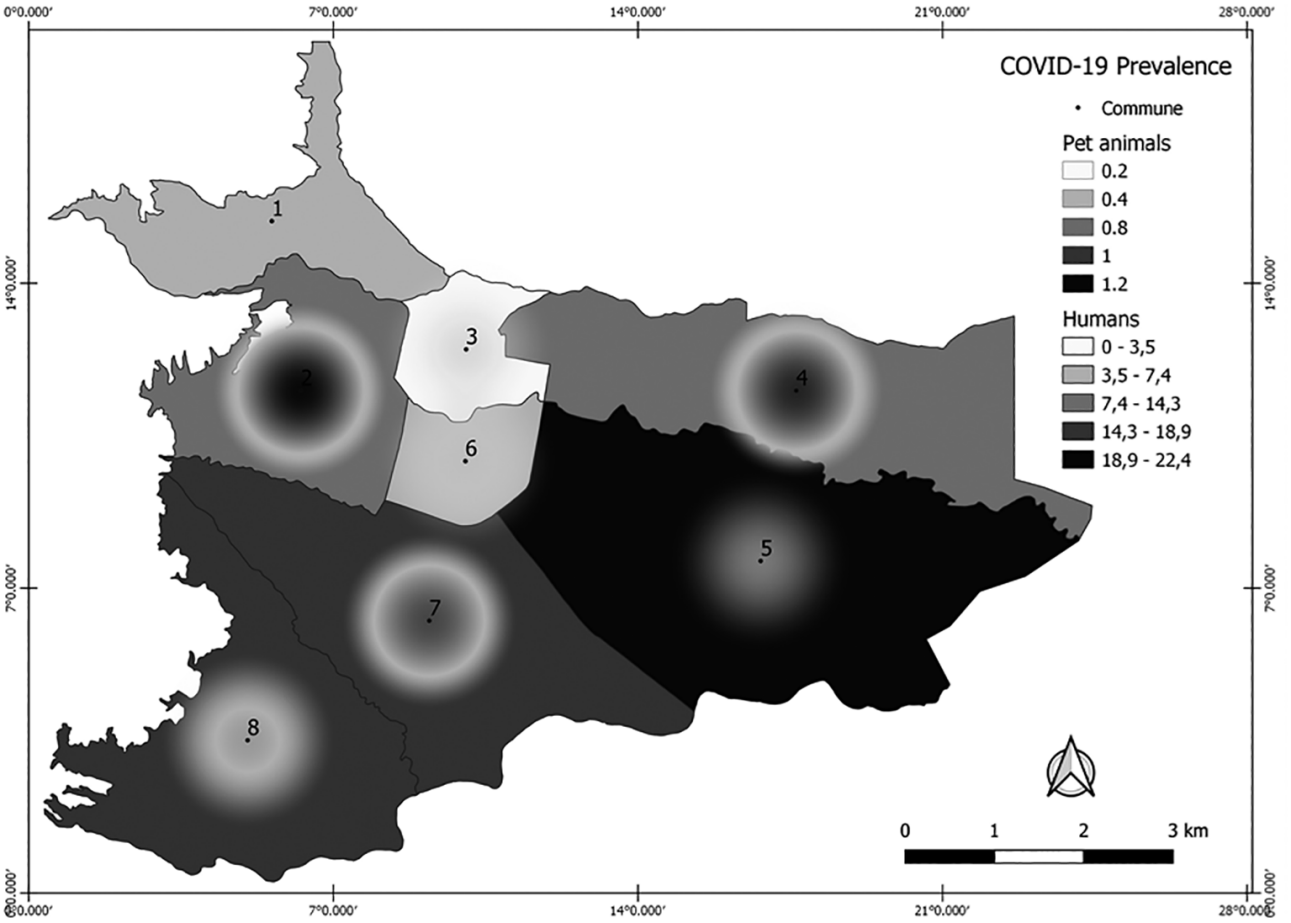
Kernel density estimation of anti-SARS-CoV-2 IgG prevalence in dogs and cats from Villavicencio. The darker shade represents the highest density of seropositive animals, and the clearest represents the sites with the lowest densities. This figure is an original figure produced by the authors for this article. COVID-19 = coronavirus disease 2019; IgG = immunoglobulin class G.

Regarding the eight communes, there was a general seroprevalence of 0, 13.63%, 6.67%, 8.06%, 1.02%, 4.76%, 4.82% and 2.5%, respectively ( Table 2). In the map obtained through Kernel density analysis ( Figure 3), it is observed that the density of cases was concentrated mainly in the west of the city. Communes with higher densities of SARS-CoV-2 seropositive animals were 2 and 4 in comparison to COVID-19 cases in humans, with a greater number of positive cases in communes 5, 7 and 8. Other visible sites of concentrations of seropositive animals, though with lower density, corresponded to communes 7 and 8. Finally, communes 1, 3, 5 and 6 had densities ranging from low to zero.

Regarding the SARS-CoV-2 exposure and the risk factors analysed, a statistically significant association between SARS-CoV-2 seropositivity and Commune 2 was found (adjusted OR = 5.84; CI 95% = 1.1-30.88). On the other hand, no significant statistical association was found ( *p* >0.05) between anti-SARS-CoV-2 IgG seropositivity and the other items assessed ( Table 2). Additionally, among the twenty seropositive animals, only one owner spontaneously confirmed to have positive RT-qPCR result for COVID-19.

Additionally, a Spearman correlation of *p* = 0.32 was found between the ratio of COVID-19 positive cases (RT-qPCR testing) of humans in November 2020 and domestic dogs and cats from the eight Villavicencio communes, result classified as a positive weak correlation. Finally, a strong positive correlation of 0.81 was found between the feline species and their SARS-CoV-2 seropositivity, as well as a positive correlation of 0.68 between the canine species and their SARS-CoV-2 seropositivity.

## Discussion

In the present study, the seroprevalence of SARS-CoV-2 in canines was 3.67% (11/300) and in felines 6.67% (9/135). Felines had more risk of becoming infected with SARS-CoV-2 that dog (adjusted OR = 2.07; 95% CI = 0.78-5.46) ( Table 2) this tendency was no statistically different. In similar studies, Barroso *et al*. ^7^ found SARS-CoV-2 seroprevalence of 4.7% in dogs and 21.7% in cats in Portugal, determining that, among seropositive animals, 50% had been possibly infected by human-animal transmission. On the other hand, 33.3% of seropositive cats had possibly been infected via the cat-cat route. Colitti *et al*. ^33^ found a SARS-CoV-2 seroprevalence of 2.3% in dogs and 16.2% in cats in Italy, and Fritz *et al.* ^13^ found a SARS-CoV-2 seroprevalence of 15.4% in dogs and 23.5% in cats from France. In all the studies mentioned, SARS-CoV-2 prevalence was higher in cats and its transmission was mostly related to exposure to humans when they were more seropositive and more susceptible to infection. ^27^^,^^33^^,^^34^

As a result of the present study, a positive relationship between seropositivity and the age of the animals was observed.The older animals between 11 and 15 years exhibited this tendency predisposition, but it was not statistically different (adjusted OR = 1.61; 95% CI = 0.16-15.93) ( Table 2). In this sense, a significant trend was found in the fatality and mortality rates of COVID-19 with advanced age in humans, ^35^ given that there is a weakened immune system, underlying chronic diseases, multiple drug therapies, lack of attention and self-care, poor environmental hygiene, loneliness, and lack of adequate support from other family members in this population. ^36^ These reasons could be considered with equal value in the case of animals, especially pet animals. ^37^ On the other hand, Shi *et al*. ^25^ reported that three-month-old canines exhibited low susceptibility to experimental infection, contrary to the results obtained in cats, since animals aged less than 100 days and up to nine months were highly susceptible to SARS-CoV-2 infection.

In the present study, no significant differences were found for respiratory and digestive symptoms of the animals sampled according to their SARS-CoV-2 seropositivity ( *X* ^2^ = 0.8206; *p* = 0.365) ( Table 3). These results are similar to those reported by Pagani *et al*. ^38^ and Shi *et al*. ^25^ i.e., cats infected with SARS-CoV-2 were asymptomatic or highly susceptible to subclinical infections. Contrarily, in Germany, Keller *et al*. ^39^ reported animals with mainly respiratory symptoms, describing the case of a cat with unresolved pneumonia, which was associated to the owner positive test for COVID-19. SARS-CoV-2-specific nucleic acid analysis was performed, revealing the complete genome and the presence of infection in that patient.

In both canines and felines, the highest seropositivity occurred in Commune 2 (13.63% [6/44]) ( Table 2), which is located in the southwest of Villavicencio. Despite the fact that it is a commune with a low population (19,491 inhabitants), ^40^ it has been reported with the highest number of inhabitants per house (6 inhabitants) in comparison to the other communes, ^41^ suggesting that having more than one individual infected with SARS-CoV-2 in the same household increased the risk of infection in these pet animals. ^33^ Likewise, this commune presented a positive association between the seropositivity of the animals sampled (adjusted OR = 5.84; 95% CI = 1.1-30.88) ( Table 2) and the seropositivity of the owners, similarly, Colitti *et al.* ^33^ found a positive association between COVID-19 positive owners and their felines’ SARS-CoV-2 seropositivity (OR = 2.5; 95% CI = 1.3-5.2), which may be related to the duration of the pets’ exposure to the infected owners, and the close contact of the felines with their owners, suggesting the development of antibodies in domestic animals as a consequence of viral transmission from owners. ^33^^,^^34^^,^^42^ In the present study, the association between positive COVID-19 cases (RT-qPCR testing) in humans versus seropositivity in canines and felines from the eight communes of Villavicencio was weakly positive (Spearman's correlation of *p* = 0.32). On the other hand, the study conducted by Van Aart *et al*. ^43^ showed that none of the felines had been infected with SARS-CoV-2 despite the fact that these were living with their positive COVID-19 owners. Therefore, these associations between species should be analysed considering different factors.

Animals and humans are susceptible to a large number of different coronaviruses, in fact, it has been shown that all pathogenic human coronaviruses have their origin in animals, which is why studies should focus on their role in the transmission of SARS-CoV-2. ^44^ In the present study, a human-animal transmission was considered based on the results of Smith *et al*. ^45^ in the United Kingdom, who ruled out that dogs and cats were reservoirs of infection for humans. However, we cannot be sure about the trasmission direction, which will only be confirmed through further studies. Ultimately, successful elimination of SARS-CoV-2 will only be possible by assessing and controlling transmission in all susceptible animal species, a one health approach that could prevent the re-emergence of the virus in the future. ^46^^,^^47^

## Conclusion

The present study provides the first positive results of anti-SARS-CoV-2 serological tests (ELISA) in domestic dogs and cats in Colombia, with information about the dynamics of virus transmission in Latin America and the world during the COVID-19 pandemic. As mentioned above, cats were more susceptible to natural SARS-CoV-2 infection than dogs, following similar dynamics described in other studies. ^7^^,^^33^^,^^48^ The present study does not provide evidence that domestic canines and felines are sources of infection for humans; however, further studies focused on one health should not be ruled out ^47^ in order to improve our knowledge about transmission, epidemiology and dynamics of SARS-CoV-2 and promote a better response to possible future pandemics.

## Data availability

### Underlying data

Figshare: Seroprevalence of exposure to SARS-CoV-2 in domestic dogs and cats and its relationship with COVID-19 cases in the city of Villavicencio, Colombia. https://doi.org/10.6084/m9.figshare.21271137. ^49^

This project contains the following underlying data:

- Dataset.xlsx (data on OD IgG Anti-SARS-CoV-2, positive control sample ratio (Indirect ELISA), data on domestic dogs and cats that participated in the project.)

### Extended data

Figshare: Seroprevalence of exposure to SARS-CoV-2 in domestic dogs and cats and its relationship with COVID-19 cases in the city of Villavicencio, Colombia. https://doi.org/10.6084/m9.figshare.21271137. ^49^

This project contains the following extended data:

- Consent form.docx
- Questionnaire.docx

Data are available under the terms of the Creative Commons Attribution 4.0 International license (CC-BY 4.0).
