# Supplementary material for: Seroprevalence of exposure to SARS-CoV-2 in domestic dogs and cats and its relationship with COVID-19 cases in the city of Villavicencio, Colombia
Source: F1000Res. 2023 Aug 10;11:1184. Originally published 2022 Oct 17. [Version 3] doi: 10.12688/f1000research.125780.3 (PMC10643872; doi:10.12688/f1000research.125780.3)
Supplement: Peer review history

# Reviewer report: Reviewer response for version 3

**DOI:** 10.5256/f1000research.152500.r218010

**Published:** 2023-11-13

**Authors:** Laura C. Bohórquez

**Affiliations:** 1 FIND, Geneva, Geneva, Switzerland

The authors have presented a prevalence study assessing the exposure to SARS-CoV-2 in domestic animals and its association to the COVID19 cases of their human owners, across 8 territorial entities in the city of Villavicencio, Colombia.

 The work is clearly presented and the authors cite relevant current literature.

 The study design is appropriate and technically sound; however, I suggest to clarify specifically what is the sample size estimated, and specify if it was estimated per commune. In the Sample size section in Methods the population assessed is referring to the anti-rabies vaccination campaign (68,651) and the formula used and parameters but it does not state the estimated “n” for this study.

 In the Statistical analysis section, please specify how the confidence intervals were calculated.

 I suggest to the authors to complement in the Methods section the minimization of bias, if it was considered before the study commence. For example, blinding test operators to the positivity status of the owners, selection of the test.

 Regarding the discussion please do not repeat all the results text again, and do not refer to the Table X or Figure Y, it is not necessary to duplicate it, that information is already detained in the Results section.

 The authors highlighted several times relationships or associations in comparisons that showed to be “not statistically significantly different”. It is not clear why, it seems they focus on the point estimates and do not consider the confidence intervals. I suggest these statements are based on the statistical measures, in that case, stating that there was not a statistical significant association or please state clearly how do you support your conclusions against the statistical evidence.

 Keeping in mind what the authors have highlighted, that this study is not assessing the transmission direction between pets and owners, the authors should be careful in the Discussion, Conclusion and Abstract sections, avoiding the statements such as that the evidence from this study show the dynamics of virus transmission. As it is not the case, I suggest to the authors to rephrase such sentences to, for example, the evidence show the association between sero-positivity in pets and COVID-19 human cases in owners.

 I suggest to the authors to complement in the Discussion section the specific limitations of the study (and potential risks of bias), this will enrich their discussion. For example, the selection of the tests, and its performance, because it seems that the performance declared by the manufacturer of the selected test is much higher for cats than for dogs and this could impact the results of this study.

 Typos:

- “if whether” in Methods
- “Dis” in Table 3
- “Ig” but it refers to IgG in Figure 2
- Spanish words in Table 4 (caninos, felinos, mascotas)

Is the work clearly and accurately presented and does it cite the current literature?

Yes

If applicable, is the statistical analysis and its interpretation appropriate?

Yes

Are all the source data underlying the results available to ensure full reproducibility?

Yes

Is the study design appropriate and is the work technically sound?

Yes

Are the conclusions drawn adequately supported by the results?

Partly

Are sufficient details of methods and analysis provided to allow replication by others?

Yes

Reviewer Expertise:

Diagnostics, Immunodiagnostics, Molecular Diagnostics, Research and Development of Diagnostics, Clinical Research of Diagnostics and Medical devices.

I confirm that I have read this submission and believe that I have an appropriate level of expertise to confirm that it is of an acceptable scientific standard.

# Reviewer report: Reviewer response for version 3

**DOI:** 10.5256/f1000research.152500.r195444

**Published:** 2023-08-15

**Authors:** Diego A. Forero

**Affiliations:** 1 School of Health and Sport Sciences, Fundación Universitaria del Área Andina, Bogotá, Colombia

The authors have incorporated my previous suggestions into the revised manuscript. I have a minor comment: content of table 4 in the current version of the manuscript is in Spanish.

Is the work clearly and accurately presented and does it cite the current literature?

Yes

If applicable, is the statistical analysis and its interpretation appropriate?

Partly

Are all the source data underlying the results available to ensure full reproducibility?

Yes

Is the study design appropriate and is the work technically sound?

Yes

Are the conclusions drawn adequately supported by the results?

Partly

Are sufficient details of methods and analysis provided to allow replication by others?

Partly

Reviewer Expertise:

Human health; molecular biology

I confirm that I have read this submission and believe that I have an appropriate level of expertise to confirm that it is of an acceptable scientific standard.

# Reviewer report: Reviewer response for version 2

**DOI:** 10.5256/f1000research.145608.r167139

**Published:** 2023-04-04

**Authors:** Diego A. Forero

**Affiliations:** 1 School of Health and Sport Sciences, Fundación Universitaria del Área Andina, Bogotá, Colombia

The authors took into account several of my previous comments (and it is evident in the revised manuscript). However, several minor aspects highlighted for me in my previous review remain to be considered in the revised manuscript:

- A better description of the rationale of the main aim of the study is needed.
- It is not clear, in the manuscript, whether the authors examined directly some of the animals, for key clinical signs.
- The statistical analysis (statistical tests used to generate a p value) in table 2 is not clear.
- Data for dogs in Figure 3 is still not clear.
- Data for correlation analyses should be presented in the manuscript (or in a supplementary file).
- In the Discussion section, authors must really keep into account the lack of statistical significance of some findings.

Is the work clearly and accurately presented and does it cite the current literature?

Yes

If applicable, is the statistical analysis and its interpretation appropriate?

Partly

Are all the source data underlying the results available to ensure full reproducibility?

Yes

Is the study design appropriate and is the work technically sound?

Yes

Are the conclusions drawn adequately supported by the results?

Partly

Are sufficient details of methods and analysis provided to allow replication by others?

Partly

Reviewer Expertise:

Human health; molecular biology

I confirm that I have read this submission and believe that I have an appropriate level of expertise to confirm that it is of an acceptable scientific standard, however I have significant reservations, as outlined above.

# Response

**Published:** 2023-07-02

**Authors:** Dumar Alexander Jaramillo Hernández

**Affiliations:** Universidad de los Llanos, Colombia

**1. A better description of the rationale of the main aim of the study is needed.**

 Change accepted:

 R/ In the last paragraph introduction:

 Colombia, in December 2021, a lion exhibited symptoms of infection days after being in contact with a COVID-19 positive keeper. 28 However, seroepidemiological studies of exposure to SARS-CoV-2 by pet animals have not been reported to date in the country. *Since the dissemination of SARS-CoV-2 in dogs and cats is weak and short-lived, anti-SARS-COV-2 antibody detection studies are the best choice to determine the circulation of this virus in these companion animals. Of course, the indirect ELISA screening tests that detect immunoglobulin anti-RBD S1 SARS-CoV-2 are called for their greater accuracy in diagnosis*.

 Reiners, N, Schnurra, C, Trawinski, H, Kannenberg, J, Hermsdorf, T, Aebischer, A, et al. Performance of a SARS CoV-2 antibody ELISA based on simultaneous measurement of antibodies against the viral nucleoprotein and receptor-binding domain. Eur J Clin Microbiol Infect Dis. (2021) 40:2645–9. doi: 10.1007/S10096-021-04284-5/TABLES/3

**2. is not clear, in the manuscript, whether the authors examined directly some of the animals, for key clinical signs.**

 Change accepted:

 R/ In the last paragraph subtitle “Sampling and inclusion criteria”

 This survey also inquired whether the individuals that cohabited with the pet animals (spontaneous communication) had histories of positive or negative RT-qPCR testing for COVID-19, and ***one of the survey questions was focused on the possibility for the owner to recognize if*** whether there were histories of clinical signs of the animals, such as signs in the upper or lower respiratory tract, or non-specific digestive signs (e.g., vomiting, diarrhoea, among others)

**3. The statistical analysis (statistical tests used to generate a p value) in table 2 is not clear.**

 R/ To determine the individual significance of each of the predictors introduced in a logistic regression model, the Z statistic and the Wald chi-test are used. Databases are included as a supplement that can be used to corroborate the data. At the epidemiological level, the confidence interval offers more information than the p value.

**4. Data for dogs in Figure 3 is still not clear.**

 Change accepted:

 R/ Figure 3 will be change.

**5. Data for correlation analyses should be presented in the manuscript (or in a supplementary file).**

 Change accepted:

 R/ The data are included as Table 4 in the article.

**6. In the Discussion section, authors must really keep into account the lack of statistical significance of some findings.**

 R/ Change accepted:

 “In the present study, the association between positive COVID-19 cases (RT-qPCR testing) in humans versus seropositivity in canines and felines from the eight communes of Villavicencio was weakly positive (Spearman's correlation of *p = *0.32). This significance may be influenced by the characterization of the survey, where due to social fear or ignorance some owners could indicate that they were not or had not been positive for the disease, while a strong positive relationship was expected, as in the case of the study conducted by Patterson et al. ^29^ in Italy. On the other hand, the study by Van Aart et al. ^43^ showed that none of the felines had been infected with SARS-CoV- despite the fact that these were living with their positive COVID-19 owners.  Therefore, these associations between species should be analysed considering different factors.”

 Only this change is made, since throughout the discussion all the results obtained are taken into account, including the data that are not statistically significant.

# Reviewer report: Reviewer response for version 1

**DOI:** 10.5256/f1000research.138120.r159713

**Published:** 2023-01-26

**Authors:** Diego A. Forero

**Affiliations:** 1 School of Health and Sport Sciences, Fundación Universitaria del Área Andina, Bogotá, Colombia

The manuscript describes an analysis of seroprevalence of anti-SARS-CoV-2 IgG in a sample of 300 dogs and 135 cats from a city in Colombia. Several major and minor issues should be addressed:

- A better description of the rationale of the main aim of the study is needed - why are you studying only seroprevalence?
- "SARS-CoV-2 seroprevalence of 9.1% (3.3% in canines and 5.8% in felines)" is not clear (it is not clear that it is additive).
- In "Villavicencio (Meta State)". "state" is not clear, in the context of the organization of Colombia.
- A better description of the recruitment strategy for the current study would be useful.
- In Methods, a better description of "commune" is needed.
- It is not clear whether the authors examined directly some of the animals, for key clinical signs.
- Key parameters, such as specificity and sensitivity, are not described for the ELISA assay used. In addition, in Methods it would be important to include citations to key previous papers using the same kit.
- In Statistical analysis, a description of the specific R packages used is needed.
- Table 2 is incomplete (the header for a group of rows is missing) and the statistical analysis in table 2 is not clear.
- Figure 2 might be presented as supplementary information.
- Figure 3 is not clear - it is not clear what is the signal for humans and what is the signal for animals; in addition authors might use colors in this figure.
- Data for correlation analyses should be presented.
- In the Discussion section, authors must keep into account the lack of statistical significance of some findings.
- Possible cross-reactivity of the ELISA assay is not discussed in the manuscript.
- Limitations of the current study and recommendations for future studies are needed.

Is the work clearly and accurately presented and does it cite the current literature?

Yes

If applicable, is the statistical analysis and its interpretation appropriate?

Partly

Are all the source data underlying the results available to ensure full reproducibility?

Yes

Is the study design appropriate and is the work technically sound?

Yes

Are the conclusions drawn adequately supported by the results?

Partly

Are sufficient details of methods and analysis provided to allow replication by others?

Partly

Reviewer Expertise:

Human health; molecular biology

I confirm that I have read this submission and believe that I have an appropriate level of expertise to confirm that it is of an acceptable scientific standard, however I have significant reservations, as outlined above.

# Response

**Published:** 2023-02-10

**Authors:** Dumar Alexander Jaramillo Hernández

**Affiliations:** Universidad de los Llanos, Colombia

1. A better description of the rationale of the main aim of the study is needed - why are you studying only seroprevalence?

 R/ One of the main limitations of our study is the lack of economic support. We could have done more advanced studies such as seroincidence, detection of neutralizing antibodies, or isolation of viral genomic material. Nevertheless, we just study seroprevalence in order to include an important and representative sample size of cats and dogs in Villavicencio.

 2. "SARS-CoV-2 seroprevalence of 9.1% (3.3% in canines and 5.8% in felines)" is not clear (it is not clear that it is additive).

 R/ Change accepted:

**Methods**

**Type of study and sample size** paragraph

 Line 2 “The sample was estimated using the formula for size by proportions in finite populations, using the results obtained by Patterson *et al*. ^29^ as a reference of p, with SARS-CoV-2 seroprevalences of 3.3% in dogs and 5.8% in cats from Italy”.

 3. In "Villavicencio (Meta State)". "state" is not clear, in the context of the organization of Colombia.

 R/ Colombia is divided into 32 departments and one capital district (Bogotá). Each Department has a capital city. In the case of the Meta Department, the capital city is Villavicencio.

 Change accepted: The word “State” will be changed for Department on each occasion.

 E.g. **Methods**

**Sampling and inclusion criteria** paragraph

 Line 1 “The participants were selected based on their mandatory participation in the 2021 rabies vaccination campaign, carried out in Villavicencio (capital of Meta department) by the health secretary”.

 However, in some publications the word “state” means “departamento”.

 4. A better description of the recruitment strategy for the current study would be useful.

 R/ The inclusion and exclusion criteria used in order to estimate the sample size are mentioned under the subheading “Sampling and inclusion criteria”.

 Change accepted:

 The subheading will change to “Sampling, inclusion, and exclusion criteria”

 The sentence “The animals that had consumed immunomodulatory medication (e.g., corticosteroid-type immunosuppressants) one week before the sampling were not included in the study.” Will be changed for:

 “We determined as exclusion criterion, the animals that had consumed immunomodulatory medication (e.g., corticosteroid-type immunosuppressants) one week before the sampling.”

 5. In Methods, a better description of "commune" is needed.

 R/ In Colombia, Departments are subdivided into municipalities which are in turn subdivided into “corregimientos” in rural areas and into “comunas” (communes) in urban areas. Each commune, in turn is divided into neighborhoods.

 Change accepted:

**Methods**

**Sampling and inclusion criteria** paragraph

 Line 2 “A probabilistic sampling was conducted by randomly selected two-stage clusters of domestic dogs and cats from the eight communes (subdivisions) that compose the urban area of Villavicencio (Figure 1), …”.

 6. It is not clear whether the authors examined directly some of the animals, for key clinical signs.

 R/ No, animals were randomly selected among those who participated in the rabies vaccination campaign. Once these were selected, a survey was applied, and one of the questions was related to the clinical signs.

 7. Key parameters, such as specificity and sensitivity, are not described for the ELISA assay used. In addition, in Methods it would be important to include citations to key previous papers using the same kit.

 R/ Change accepted:

**Methods**

**Immunoassay **paragraph

 Line 1. IgG antibodies against the nucleocapsid protein (N) of SARS-CoV-2 in the sera of domestic dogs and cats were qualitatively determined using the indirect enzyme-linked immunosorbent assay (ELISA) (ID Screen ^®^ SARS-CoV-2, double antigen multi-species [IDvet, Grabels, France]) according to the manufacturer's instructions. For cats, the kit presents 63% of sensitivity and 96% of specificity. For dogs, the kit has 36% of sensitivity and 85% of specificity. Previous papers used the ID Screen ^® ^kit in their studies (48-51).

**References**

 48. Bessière P, Vergne T, Battini M, Brun J, Averso J, *et al*.: **SARS-CoV-2 Infection in Companion Animals: Prospective Serological Survey and Risk Factor Analysis in France**. *Viruses*. 2022; **14**(1178). PubMed Abstract I Publisher Full Text

 49. Diezma-Díaz C, Álvarez-García G, Regidor-Cerrillo J, Miró G, Villanueva-Saz S, *et al*.: **A comparative study of eight serological methods shows that spike protein-based ELISAs are the most accurate tests for serodiagnosing SARS-CoV-2 infections in cats and dogs**. *Front. Vet.* 2023. Sci. **10**(1121935). Publisher Full Text

 50. Barua S, Hoque M, Adekanmbi F, Kelly P, Jenkins-Moore M, *et al*.: **Antibodies to SARS-CoV-2 in dogs and cats, USA**. *Emerg Microbes Infect.* 2021. **10**(1):1669-1674. PubMed Abstract I Publisher Full Text

 51. Neira V, Brito B, Agüero B, Berrios F, Valdés V, *et al*.: **A household case evidences shorter shedding of SARS-CoV-2 in naturally infected cats compared to their human owners**. *Emerg Microbes Infect*. 2021. **10**(1):376-383. PubMed Abstract I Publisher Full Text

 8. In Statistical analysis, a description of the specific R packages used is needed.

 R/ Change accepted:

**Methods**

**Statistical analysis **paragraph

 Line 9. Statistical estimates were made using the R 4.2 software with the packages dplyr, MASS, corrplot and epiDispaly, and the maps using the QGIS 3.10 software.

 9. Table 2 is incomplete (the header for a group of rows is missing) and the statistical analysis in table 2 is not clear.

 R/ In theTable 2, the header of the row 1, 2, 3, 4 … 8: is “Communes”. This header is included in the original manuscript.

 On the other hand, statistical analysis included in table 2 are described in the fourth paragraph of results:

 Line 1 “Regarding the SARS-CoV-2 exposure and the risk factors analysed, a statistically significant association between SARS-CoV-2 seropositivity and Commune 2 was found (adjusted OR = 5.84; CI 95% = 1.1-30.88).”

 10. Figure 2 might be presented as supplementary information.

 R/ Change not accepted, it is important to show the OD distribution among communes, showing the positive results and grey area results.

 11. Figure 3 is not clear - it is not clear what is the signal for humans and what is the signal for animals; in addition authors might use colors in this figure.

 R/ Change accepted:

**Results**

 Figure 3 will be change by the colored one. 

 12. Data for correlation analyses should be presented.

 R/ Change not accepted, here we present a table with the data for the correlation analyses about the association between positive COVID-19 cases (RT-qPCR testing) in humans versus seropositivity in pets. Nevertheless, it is not necessary to show the table in the paper, since we mention the important data in the discussion (paragraph 4, line 10).

                       Human   Dogs    Cats    Pets

 Dogs                0,01         1         --          --

 Cats                0,33       0,17      1

 Pets                 0,32      0,68    0,81*       1

 * *p*-value = 0.01467

 13. In the Discussion section, authors must keep into account the lack of statistical significance of some findings.

 R/ In the results and discussion sections, we mentioned and pointed out that results were not significant (just the results associated with commune 2 were statistically significant); however, there were trends that correlate with other studies and we mentioned them explaining that these were not significant (Eg. Discussion paragraph 2, line 1):

 “As a result of the present study, a positive relationship between seropositivity and the age of the animals was observed. The older animals between 11 and 15 years exhibited this tendency predisposition, but it was not statistically different”.

 14. Possible cross-reactivity of the ELISA assay is not discussed in the manuscript.

 R/ Change accepted: The following paragraph will be added to the discussion section.

**Discussion**

 Although the 20 canine pre-pandemic sera reacted negatively to the immunodiagnosis, cross-reactions with ancestral coronaviruses in canines and felines are possible, but in low probability when are compared to commercial ELISAs with neutralizing antibody tests (54, 55).

 54. Perera R, Ko R, Tsang O, Hui D, Kwan M *et al*.: **Evaluation of a SARS-CoV-2 surrogate virus neutralization test for detection of antibody in human, canine, cat, and hamster sera.** *J Clin Microbiol*. 2021;59(2):e02504–20. PubMed Abstract I Publisher Full Text

 55. Hughes EC, Amat JAR, Haney J, Parr J, Logan N, *et al*.: **SARS-CoV-2 serosurveillance in a patient population reveals differences in virus exposure and antibody-mediated immunity according to host demography and healthcare setting**. *J Infect Dis*. 2021;223(6):971-980. PubMed Abstract I Publisher Full Text

 15. Limitations of the current study and recommendations for future studies are needed.

 R/ Change accepted: The following paragraph will be added to the discussion section.

**Discussion**

 The possible cross-reactivity and the need to verify if the reactive antibodies are neutralizing for SARS-CoV-2 are the main limitations of our study; likewise, it is advisable to carry out studies of SARS-CoV-2 neutralization from the serum bank obtained, since this is the gold-standard test for the Centers for Disease Control and Prevention (CDC) (56).

 56. Centers for Disease Control and Prevention. **Coronavirus disease 2019 (COVID-19) 2020 interim case definition, approved April 5, 2020.** 2023 [cited 2023 February 9]. Reference Source
